# Female cortical cellular mosaicism underlies shared MeCP2 and PCB impacted gene pathways

**DOI:** 10.1016/j.isci.2026.115573

**Published:** 2026-04-20

**Authors:** Osman Sharifi, Kari E. Neier, Anthony Valenzuela, Christina G. Torres, Ian Korf, Pamela J. Lein, Dag H. Yasui, Janine M. LaSalle

**Affiliations:** 1 Medical Microbiology and Immunology, School of Medicine, University of California, Davis, Davis, CA 95616, USA; 2 Cellular and Molecular Biology, College of Biological Sciences, University of California, Davis, Davis, CA 95616, USA; 3 Genome Center, University of California, Davis, Davis, CA 95616, USA; 4 MIND Institute, University of California, Davis, Davis, CA 95616, USA; 5 Department of Molecular Biosciences, Weill School of Veterinary Medicine, University of California, Davis, Davis, CA 95616, USA

**Keywords:** health sciences, neuroscience, molecular neuroscience, systems neuroscience, cellular neuroscience, systems biology

## Abstract

Rett syndrome (RTT) is an X-linked, dominant neurodevelopmental disorder caused by mutations in *MECP2*, encoding the epigenetic regulator methyl CpG binding protein. Variability in severity and timing of progression in RTT, influenced by factors including mutation type, genetic background, and X chromosome inactivation patterns, suggests potential interaction with environmental neurotoxicants such as lipophilic polychlorinated biphenyls (PCBs). To understand shared mechanisms, we exposed WT and *Mecp2e1*^−/+^ female mice to a human-relevant PCB mixture and dose, then performed single-nucleus 5′ RNA-seq from cortex. We identified significant overlap in dysregulated genes and 71 shared pathways between the effects of PCB exposure and MeCP2 mutation, and co-mitigation of their transcriptional impacts. PCBs influenced the non-cell-autonomous transcriptional effects of MeCP2 mutations in wild-type-expressing neurons within the mosaic mutant female cortex in both mouse and human, suggesting that the interactions predominantly involve homeostatic gene networks. These findings have broader implications for gene by environment interactions in neurodevelopment.

## Highlights

• PCBs and *Mecp2* mutation converge on 71 shared dysregulated KEGG pathways
• PCBs primarily affect WT-expressing neurons in the mosaic RTT female cortex
• PCB-*Mecp2* transcriptional interactions are conserved in mouse and human cortex
• GABAergic Sst neurons show strongest gene-by-environment interaction effects

## Graphical abstract

**Figure:**
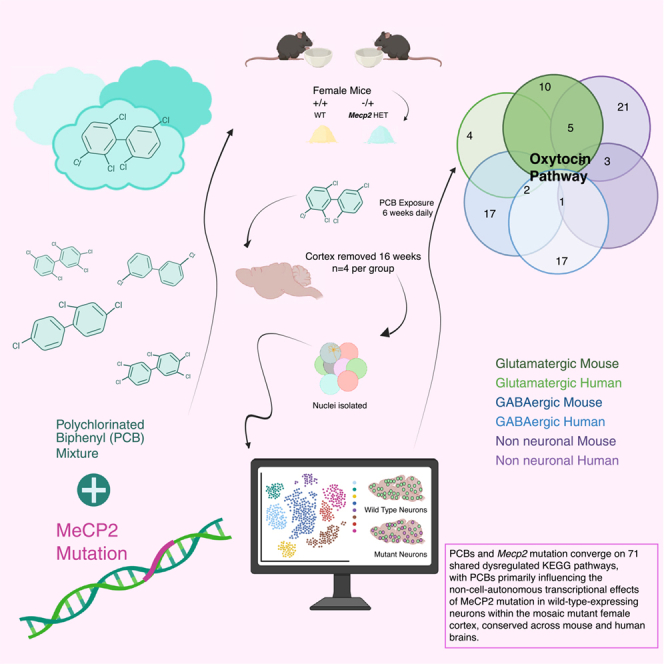

## Introduction

In female mammals, X chromosome inactivation (XCI) is random, affecting either the maternal or paternal allele in each cell. Once established, this creates a cellular mosaicism throughout the body, unless there is selective loss of one pattern.^1^ Rett syndrome (RTT) is an X-linked dominant neurodevelopmental disorder that primarily affects females and is caused by mutations in *MECP2*.^2^ This gene encodes methyl CpG binding protein 2 (MeCP2), a critical epigenetic regulator that modulates gene expression by binding to methylated DNA and acting as either a repressor or activator of transcription.^3^^,^^4^^,^^5^ RTT typically occurs after an initial period of normal development, with regression of cognitive and motor skills during late infancy, followed by several subsequent stages of regression through young adulthood, highlighting the importance of MeCP2 for the maintenance of normal neurological function.^6^

The progression and severity of RTT are highly variable among affected individuals. This phenotypic variability arises from multiple well-established factors: (1) the specific type and location of *MECP2* mutations, which can differentially impact protein function^6^; (2) individual genetic background differences that modify disease expression^7^; and (3) patterns of X chromosome inactivation (XCI) skewness, where non-random inactivation can result in varying proportions of cells expressing mutant versus wild-type MeCP2.^8^ While these intrinsic genetic and epigenetic factors are primary determinants of RTT severity, the potential contribution of environmental factors to phenotypic variability remains understudied.

The relevance of investigating MeCP2-environment interactions extends beyond RTT specifically. Although RTT is a relatively rare disorder, MeCP2 dysregulation has broader implications for neurodevelopmental disorders. Notably, reductions in MeCP2 protein levels have been documented in approximately 70% of postmortem brain samples from individuals with autism spectrum disorder (ASD).^9^ Furthermore, X-linked genes, including those involved in chromatin regulation, are significantly enriched among ASD-associated mutations.^10^ Therefore, understanding gene-by-environment (GxE) interactions in the context of MeCP2 dysfunction using the RTT model provides a tractable experimental system for elucidating mechanisms that may be applicable to non-syndromic forms of ASD and other neurodevelopmental conditions where epigenetic regulation is compromised.

Among environmental factors that could contribute to neurodevelopmental variability, persistent organic pollutants (POPs), particularly polychlorinated biphenyls (PCBs), represent candidates of significant public health concern due to their environmental presence despite regulatory restrictions.^11^^,^^12^ PCB 95, in particular, has been shown to promote dendrite growth and synaptogenesis, leading to dysregulated neuronal connectivity.^13^^,^^14^ Importantly, exposure to PCBs varies within human populations. Studies of the ASD enriched-risk Markers of Autism Risk Learning Early Signs (MARBLES) cohort and other populations have documented considerable heterogeneity in PCB congener levels, with maternal serum concentrations ranging from 0 to 37.83 ng/g lipid for total PCBs^11^^,^^14^ (Figure S5). This variability in exposure levels supports the biological plausibility that PCBs could interact with genetic susceptibility factors to influence phenotypic outcomes in neurodevelopmental disorders. We previously examined the differentially methylated regions (DMRs) of fetal brain and placenta of mice exposed to PCBs and revealed a significant overlap between MeCP2-regulated genes and differentially methylated genes resulting from prenatal PCB exposure.^15^

PCBs have been detected in the brains of individuals with various NDDs, including RTT.^11^ Research has demonstrated that exposure to PCB 95 can alter the DNA methylation patterns of over 1,000 genes in human neuronal cells, suggesting a potential interaction between genetic defects and environmental exposures in the disruption of chromatin epigenetics.^16^ Furthermore, the MARBLES study has measured PCB congeners in mothers at increased likelihood for ASDs, highlighting the relevance of PCB exposure in developmental neurotoxicology.^17^ Exposing mice to a mixture of the PCB congeners identified in the MARBLES study led to an increase in dendritic branching of cortical neurons^18^ and significant deficits in sociability and ultrasonic vocalizations, as well as enhanced repetitive behavior specifically in males at the lowest dose tested (0.1 mg/kg/d).^19^

We previously described the characterization of *Mecp2e1^-^*^-^/+^^ mutant mouse model of RTT, modeled after a human mutation, recapitulates the RTT-relevant extended period of disease symptom progression. Behavioral and physiological phenotypes in *Mecp2e1*^*−/+*^ females are detectable as early as 2.5 months of age, including motor deficits, altered social behavior, and metabolic changes.^7^^,^^20^^,^^21^ Using single nucleus RNA sequencing (snRNA-seq 5′), we recently demonstrated a dynamic, non-cell autonomous effect in wild-type expressing cells within the cortex of this construct-relevant model of RTT that corresponded with disease progression.^21^ Six times more DEGs were observed in *Mecp2e1*^*−/+*^ females than *Mecp2e1*^*-/y*^ males. Unlike males, female transcriptional dysregulation appeared prior to symptoms, were enriched for homeostatic gene pathways in distinct cell types over time, and correlated with disease phenotypes.^21^

To explore the intersection of genetic and environmental factors in RTT female X chromosome mosaicism, we utilized a construct-relevant mouse model of RTT which carries a mutation based on a human RTT patient mutation and performed snRNA-seq 5′ on cortical cell types from adult female mice exposed to the MARBLES PCB mix. We demonstrate a highly significant overlap in dysregulated genes and molecular pathways and between the effects of PCB exposure and *Mecp2* mutations. Interestingly, PCBs preferentially impact the non-cell-autonomous transcription effects of *Mecp2* mutation in wild-type-expressing neurons within *Mecp2* mosaic mutant female cortex, suggesting the interaction is predominantly with homeostatic gene networks. By establishing a baseline for single-cell gene expression in PCB-exposed mice, our study aims to elucidate the complex interplay between genetic and environmental factors in RTT and related neurodevelopmental disorders, providing insights into how PCB exposures may modulate disease progression and severity.

## Results

### Experimental design to test transcriptional dysregulation in PCB-exposed WT and *Mecp2e1* heterozygous female cortical cell types

**Figure 1:**
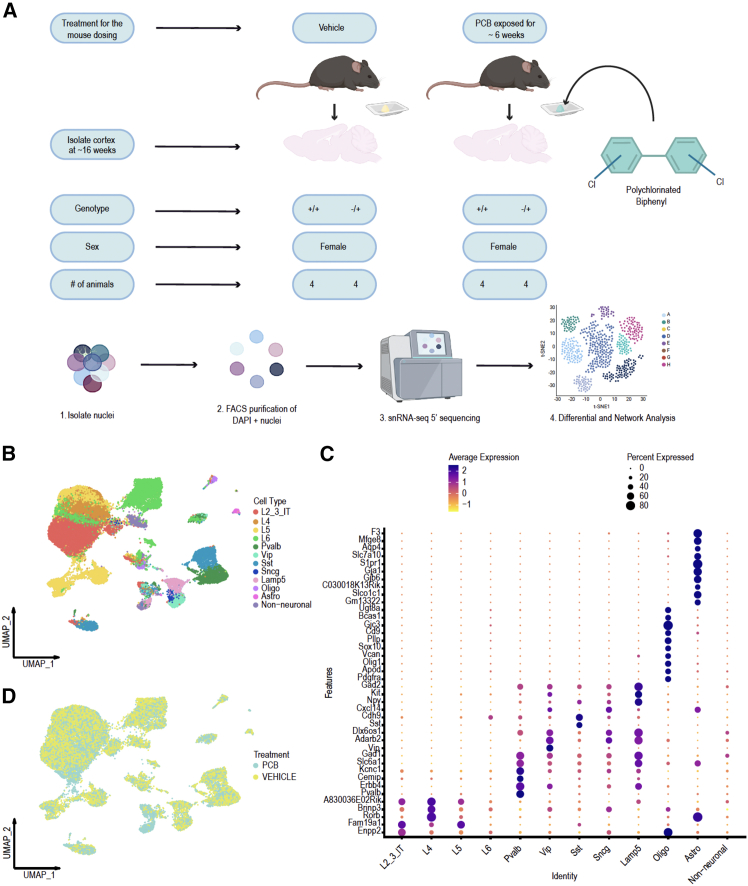
Single nuclear transcriptome study design to investigate effects of *Mecp2* mutation and PCB exposure (A) Schematic showing cortical samples from PCB or vehicle treatment groups of two genotypes (*Mecp2e1*^*+/+*^ WT or *Mecp2e1*^*−/+*^ HET) for snRNA-seq 5′. (B) UMAP unsupervised clustering of cell types identified in cortex from all samples. (C) Dot plot of the top marker genes for each cell type identified. Color intensity indicates average scaled expression (*Z* score normalized across genes); dot size represents percentage of cells expressing each gene within each cell type. (D) UMAP plot of cell cluster by treatment group.

To identify cell type and PCB exposure specific transcriptional differences in *Mecp2e1* deficient mouse cortex, we performed single nucleus RNA sequencing (snRNA-seq 5′) on late disease stage *Mecp2e1*^*−/+*^ (∼16 weeks of age) heterozygous (HET) females and sex-matched wild-type *Mecp2e1*^*+/+*^ (WT) littermates (Figure 1A). Based on Sharifi 2024, we expect DEGs in females to be most prominent at this symptomatic disease stage when early symptoms are emerging in this model.^21^ Female mice of each genotype were orally exposed to either the PCB MARBLES mix or vehicle control daily for ∼6 weeks for a total of 16 animals (4/group) (Figure 1A). To elucidate the cellular landscape within the cortices of WT and HET mice, we applied unsupervised clustering analysis to classify cells based on their gene expression profiles and labeled cell types based on the Allen Brain Atlas cortex ref.^22^ We identified 12 distinct cell types within the cortical tissue (Figure 1B). Cell type identification and labeling was validated based on marker genes present in each cell type (Figure 1C). Each marker was selected based on its known association with specific cell types and validated the accuracy of our clustering results. To assess the influence of PCB exposure on cell type clustering, we compared the clustering results of cells treated with the PCBs to those treated with a vehicle control (Figure 1D). Cells from both treatment conditions were clustered mostly together, and the resulting cell type distributions were analyzed for differentially expressed genes (DEGs).

### Cell type stratified differential gene expression (DEG) analysis of cell types in PCB-treated versus vehicle-treated females of both *Mecp2e1* genotypes

**Figure 2:**
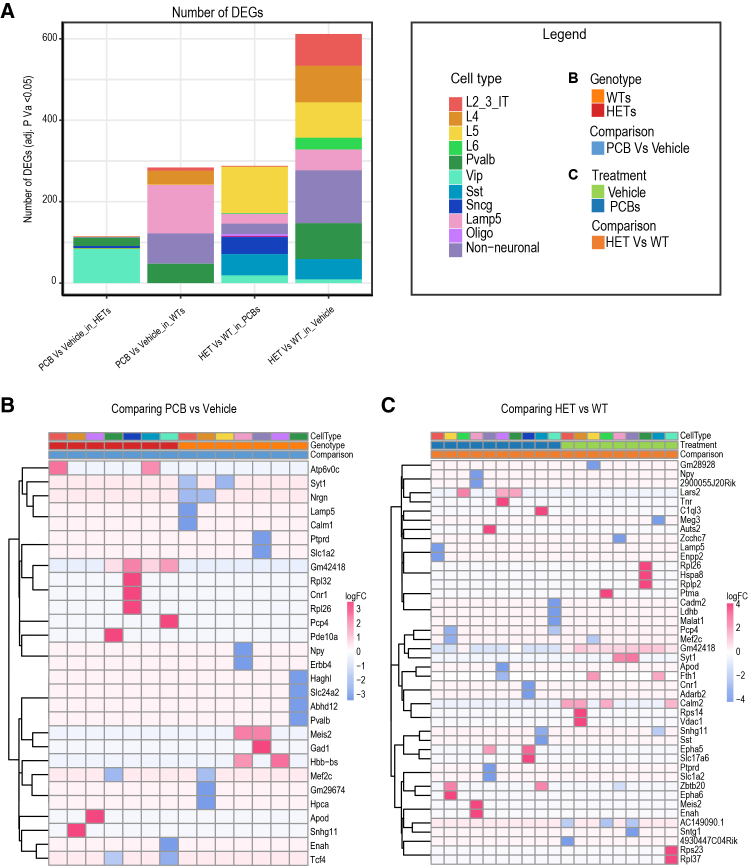
Interaction between *Mecp2* genotype and PCB exposure in frequency and cell type distribution of DEGs (A) Bar graph showing the significant number of DEGs for each experiment. (B and C) Heatmaps showing the top DEGs by log-fold change (adjusted *p* value ≤ 0.05) per cell type for comparing PCB vs. vehicle and comparing *Mecp2e1*^−/+^ HET vs. WT cortices respectively. Positive log2 fold changes (red) indicate upregulation in PCB-exposed or HET mice; negative values (blue) indicate downregulation. Genes were selected by ranking all significant DEGs by absolute logFC within each comparison and displaying the top-ranked genes. (B) Comparing HET vs. WT cortices; (C) comparing PCB vs. vehicle exposure.

To further explore the impact of PCB exposure on gene expression within distinct cell types, we performed a differential expression analysis comparing vehicle-treated and PCB-treated conditions in both WT and HET mice. This analysis aimed to identify changes in gene expression associated with PCB exposure and evaluate potential interaction effects of PCB treatment in the context of RTT HET genotype (Figure 2). To ensure DEGs were not driven by changes in cellular proportions, we conducted statistical analysis of cell type proportions across all four experimental conditions (Figure S1; Table S1). While some cell types showed variation across conditions, these differences did not reach statistical significance after correction for multiple comparisons, consistent with previous findings.^23^ To further control for any potential effects of cell type proportion variability, we downsampled cells to ensure equal representation across conditions before performing differential expression analysis. We first compared cell type DEGs between WT and HET mice within the vehicle-treated and PCB-treated backgrounds. This comparison aimed to discern how PCB exposure interacts with genetic differences between WT and HET mice. The variability in number of DEGs and expression patterns across different cell types underscores the complexity of PCB’s effects, which may vary depending on the specific cellular context and the nature of the gene expression changes (Figure 2A). The highest number of DEGs was observed between WT and HET cortical cells with vehicle treatment. Conversely, PCB-treated HET mice showed a lower number of DEGs compared to vehicle-treated HET cortical cells (Figure 2A). These findings highlight a potential modulatory role of PCB in the context of HET, where PCB treatment may normalize or attenuate some of the transcriptional disruptions caused by the *Mecp2e1* mutations. These DEGs are primarily associated with neuronal function, brain development, signaling pathways, and metabolism (Figures 2B and 2C). Many DEGs encode proteins in the nervous system, including neurotransmitter regulation such as *Gad1* and *Slc1a2*, calcium signaling (*Calm2* and *Hpca*), and transcription factors involved in development (*Zbtb20* and *Meis2*) (Figures 2B and 2C). Comparisons of PCB-treated vs. vehicle and WT cortices vs. RTT cortices both contain genes involved in neuronal signaling and synaptic function (*Syt1*, *Nrgn*, *Calm1/Calm2*, *Cnr1*, *Slc1a2*, and *Pcp4*), suggesting a dysregulation of neuronal communication.

### Commonly altered KEGG pathways are observed across experimental conditions

**Figure 3:**
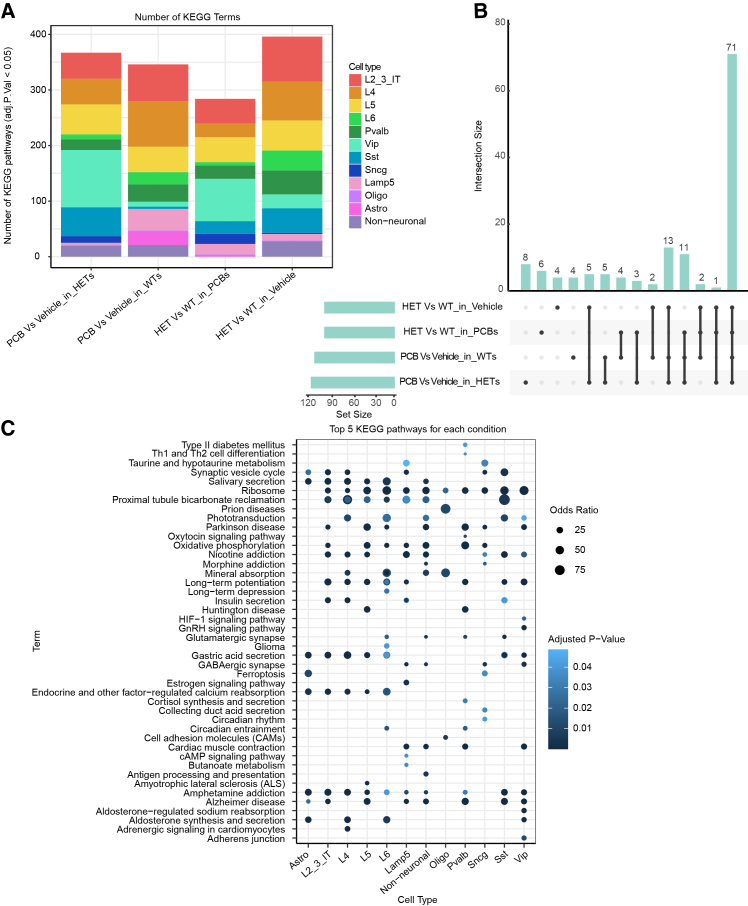
KEGG pathway analysis shows convergent biological pathways between *Mecp2* genotype and PCB exposure (A) Bar graphs showing the number of significant dysregulated KEGG pathways per cell type. (B) Upset plot showing the number of significant pathways overlapping across the four experimental pairwise comparisons. (C) Dot plot showing KEGG pathways that were significantly enriched across the four experimental comparisons shown in (B) (WT vs. HET in vehicle; WT vs. HET in PCB; vehicle vs. PCB in WT; vehicle vs. PCB in HET). Pathways displayed were selected if they ranked among the top 5 most significantly enriched (by adjusted *p* value) in at least one cell type. Because different cell types have different top-ranked pathways, and some pathways appear in multiple cell types, more than 20 unique pathways are shown. Dot size represents the odds ratio of enrichment; color intensity represents the adjusted *p* value (Fisher’s exact test with Benjamini-Hochberg correction). Each dot represents the enrichment statistics for that pathway in that specific cell type, with data aggregated across all four pairwise comparisons.

To understand how PCB exposure affects cellular pathways, we analyzed dysregulated KEGG pathways across four pairwise comparisons of cell type DEGs: WT vs. HET in vehicle, WT vs. HET in PCB, vehicle vs. PCB in WT, and vehicle vs. PCB in HET. This analysis aimed to identify common and unique pathway alterations associated with the interaction between PCB exposure and heterozygous *Mecp2e1* mutation. While the total number of KEGG pathways did not vary by comparison group, non-neuronal cell types showed a reduction in KEGG terms specifically in the genotype comparison within PCB-treated animals (Figure 3A). A closer look at the dysregulated pathways reveals that the majority of these KEGG pathways are highly similar across all four group comparisons, with 71 overlapping pathways (Figure 3B). This extensive overlap indicates that both PCB exposure and *Mecp2e1* genotype converge on a core set of dysregulated gene pathways. Across four experimental conditions, these overlapping dysregulated gene pathways were more impacted in neuronal than glial cells (Figure 3C; Table S2). Further, the KEGG pathways that were dysregulated across conditions and cell types include those related to neuronal and synaptic function such as GABAergic synapse, glutamatergic synapse, synaptic vesicle cycle, long-term potentiation (LTP), long-term depression (LTD), circadian rhythm and circadian entrainment, amphetamine addiction, morphine addiction, and nicotine addiction (Figure 3C). These neurologically relevant pathways underscore the potential interaction of PCB exposure and a neurodevelopmental disorder mutation on key aspects of neuronal function and regulation. In addition, many of the dysregulated paths are related to neurogenerative diseases such as Parkinson’s disease and Alzheimer’ disease, which is consistent with previous studies.^24^^,^^25^

### Investigating PCB effects on *Mecp2e1* heterozygous mosaic cortices and cell non-autonomous interactions

To accurately distinguish between wild-type (WT) and mutant *Mecp2*-expressing cells within a mosaic female brain, we developed a bioinformatic pipeline (Figure S2). This pipeline integrates preprocessing, feature selection, dimensionality reduction, clustering, and classification to achieve precise segregation of WT and mutant *Mecp2*-expressing cells in the mosaic brain in the context of our gene x environment experimental design.

**Figure 4:**
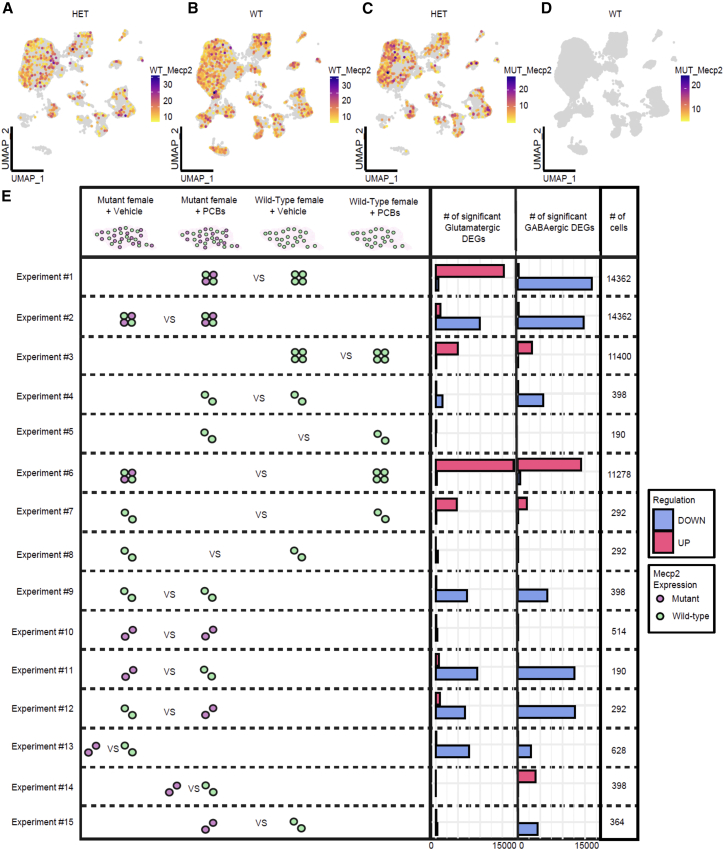
Mosaic cellular parsing reveals PCB effects on non-cell-autonomous transcriptional dysregulation in RTT mouse model (A–D) UMAP plots showing WT_*Mecp2* and MUT_*Mecp2* expression in WT and *Mecp2e1* mutant heterozygous cortices. (E) DEG experiments comparing WT (green) or mutant (purple) single cells across sample types in glutamatergic and GABAergic cells. Fifteen experimental comparisons (*y* axis) for DEGs (bar graphs; red, up; blue, down for right group) revealed opposite PCB effects on glutamatergic vs. GABAergic neurons in bulk HET (#1) and opposite effects of mutant-expression within HET vs. WT cortical GABAergic neurons (#14–15). No mutant-expressing cells were detected in WT cortex, as expected. Cells were downsampled to the lowest number of cells per pair, *n* = 3 mice/group.

To investigate how PCB exposure affects WT-*Mecp2* versus mutant-expressing cells within female mosaic cortical tissue, we first parsed *Mecp2* transcript genotypes in all cells (Figures 4A–4D). As expected, no mutant cells were observed in WT cortices (Figure 4D), and female heterozygous cortical cells were a roughly equal mixture of WT- and mutant-expressing (Figures 4A and 4C).

To reduce the impact of lower cell counts on DEG calling following parsing, we further grouped the *Mecp2* expressing cells into neuronal categories of GABAergic and glutamatergic neurons to gain insight into cell non-autonomous effects. The number of cells analyzed are given for each comparison after downsampling so that the pairwise comparisons were equal (right column Figure 4E; Table S3). Non-neuronal cells were not sufficiently abundant to allow for DEG analyses after parsing. To evaluate the effects of *Mecp2* genotype and PCB exposure on neuronal populations, we performed 15 distinct pairwise differential expression (DEG) comparisons using the same pool of cells from all 16 animals, each designed to interrogate specific aspects of gene-by-environment interactions in the mosaic brain (Figure 4E). Experiments #1, 2, 3, and 6 are from non-parsed (4 dot) glutamatergic or GABAergic neurons for comparison to parsed (2 dot) neurons in remaining experiments that were either WT-expressing (green) or mutant-expressing (purple). Interestingly, a comparison of PCB effects in WT-expressing cells compared to mutant-expressing cells in HET (experiments 9 and 10) showed a higher number of DEGs in WT-expressing neurons than mutant-expressing neurons of both types. Remarkably, the lowest numbers of DEGs were seen when comparing WT-expressing neurons across both genotype and treatment groups (experiments #5 and 8) or mutant-expressing neurons with or without PCBs (experiment #10). This indicates that PCB exposure predominantly affects *Mecp2* WT-expressing neurons, suggesting that PCB interaction effects within RTT cortices are predominantly acting on the homeostatic gene networks dysregulated in WT-expressing neurons. Comparisons of non-parsed neurons within HET mice with or without PCB (experiments #1 and #2) showed that the number and direction of DEGs were different between excitatory and inhibitory neurons only in the presence of PCBs. Collectively, these results suggest that PCB treatment interacts with and could mitigate some of the detrimental non-cell-autonomous effects caused by *Mecp2* mutation (Table S4). The differences observed in DEG counts between *Mecp2e1* WT- and mutant-expressing cells, and the impact of PCB treatment on these cells, highlight the complex interactions within the mosaic brain environment. PCB exposure appears to interact with *Mecp2e1* mutation by transcriptionally dysregulating shared homeostatic pathways regulating the broader cellular milieu, demonstrating a nuanced interaction between a genetic and environmental factor.

### Network analysis using hdWGCNA associates PCB and *Mecp2e1* genotype effects with RTT mouse model phenotypes

**Figure 5:**
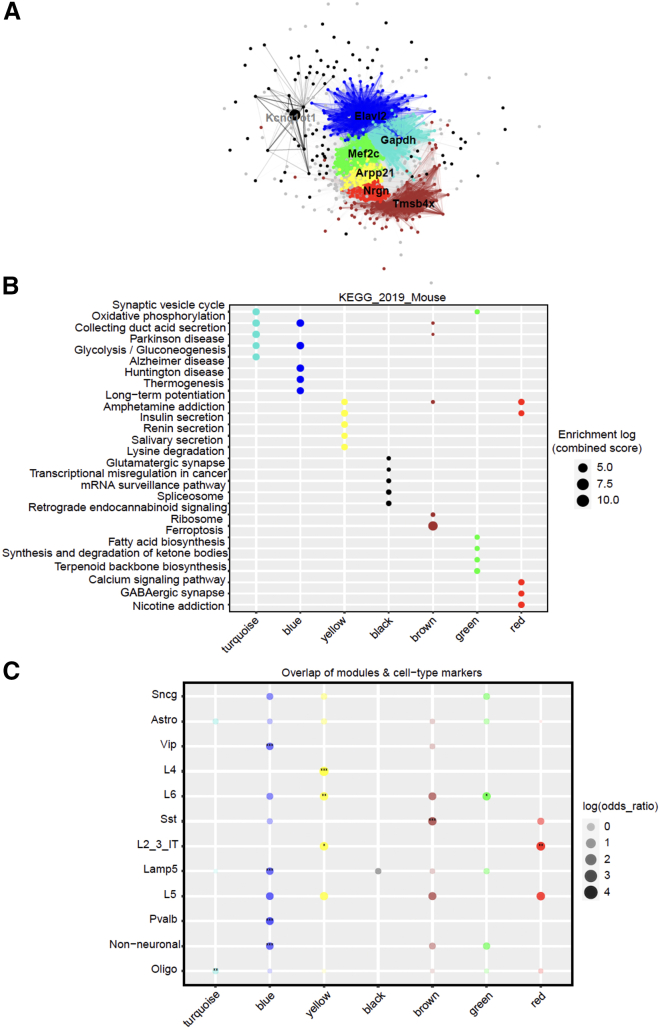
hdWGCNA analysis reveals correlated transcriptional networks impacted by *Mecp2e1* mutation and PCBs Modules were constructed using cells from all four experimental conditions (WT_Vehicle, WT_PCB, HET_Vehicle, and HET_PCB) to identify co-expression networks, then correlations were examined to determine associations with genotype (HET vs. WT) and treatment (PCB vs. vehicle). (A) Weighted gene network showing seven modules and their corresponding hub genes. (B) Dot plot showing the top 5 KEGG pathways enriched in each module. (C) Dot plot showing the overlap of cell type markers and gene modules. Module-trait correlations showing associations between each module and experimental variables (PCB treatment and Mecp2 genotype) are provided in Figure S3 and Table S5.

To further explore the effects of *Mecp2e1* mutations and PCB exposure on RTT phenotypes, we employed high dimensional weighted gene co-expression network analysis (hdWGCNA). This approach is relevant for assessing gene networks within snRNA-seq data and allowed us to analyze associations of experimental variables (treatment, genotype, exposure duration, and pregnancy) and measured phenotype (body weight at time of sacrifice at ∼16 weeks) with gene co-expression networks. Using cells from all four experimental conditions combined (WT_Vehicle, WT_PCB, HET_Vehicle, and HET_PCB), hdWGCNA analysis identified seven distinct co-expression modules (Figure 5A). Each module represents a cluster of co-expressed genes with common functional characteristics. Hub genes within each module are highlighted, serving as central connectors that are crucial for the module’s function and overall network stability (Figure 5A). The genes from these distinct modules were used to perform KEGG pathway enrichment analysis. The top KEGG pathways for each module reflect the biological processes and pathways most prominently affected by PCB exposure and/or *Mecp2e1* genotype (Figure 5B). These pathways include many neurological disease related terms such as Parkinson’s disease, Alzheimer’s disease, calcium signaling, and LTP which provide insights into the functional impact of these factors on gene networks (Figure 5B). To confirm that the gene networks are not influenced by cell type-specific gene markers, we performed an overlap analysis between cell type gene markers and the identified gene modules (Figure 5C). Additionally, we examined the correlation of gene expression in each cell type with various experimental variables, including pregnancy, weight at sacrifice, exposure duration, PCB treatment, and genotype (Figure S3; correlation values in Table S5). Among the different cell types analyzed, the Sst inhibitory neurons showed the strongest correlations with changes in gene expression linked to genotype and phenotypic traits, suggesting that *Mecp2e1* genotype and PCB exposures interact within shared excitatory/inhibitory balance and homeostatic transcriptional networks in the female adult cortex.

### Comparative analysis of mouse and human RTT cortex PCB-associated DEGs and enriched pathways in GABAergic, glutamatergic, and non-neuronal cells

**Figure 6:**
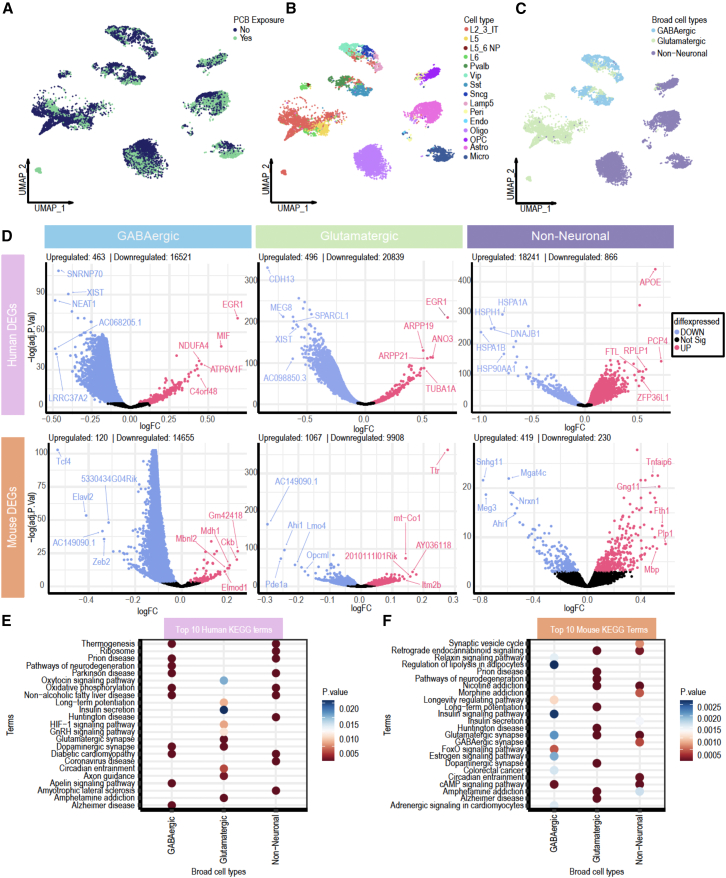
Comparative analysis of mouse and human RTT cortex PCB-associated DEGs in GABAergic, glutamatergic, and non-neuronal cells (A) UMAP plot of human postmortem cortical nuclei showing PCB and non-PCB exposed cell clustering. (B) UMAP plot of human postmortem cell type annotation. (C) UMAP plot of human postmortem broad cell type categories. (D) Volcano plots showing differential gene expression comparing PCB-exposed versus non-PCB-exposed in human and mouse within each broad cell type category. Genes with adjusted *p* value ≤ 0.05 are colored (red = upregulated in PCB-exposed; blue = downregulated in PCB-exposed; black = not significant). (E) Dot plot showing top 10 enriched KEGG pathways in human PCB-exposed tissue across broad cell types. (F) Dot plot showing top 10 enriched KEGG pathways in mouse PCB-exposed tissue across corresponding broad cell types. Dot size represents enrichment odds ratio; color intensity represents adjusted *p* value.

To evaluate the relevance of our findings in a mouse model to human RTT, we compared cell type-specific DEGs associated with PCB exposures between human female RTT cortex samples with measured PCB levels^11^^,^^21^ (Figure S5) and mouse female HET cortex samples. Figure 6 illustrates this comparative analysis, highlighting conserved and divergent molecular signatures across species. UMAP projection of human RTT brains, stratified by PCB exposure status (Yes means any PCB congener measured above level of detection), revealed similar cell clustering associated with exposure (Figure 6A). Human cell types were identified and annotated based on the Allen Brain Human Cortex dataset^26^ (Figure 6B). For a cross-species comparative analysis, these cell types were grouped into three major categories: GABAergic neurons, glutamatergic neurons, and non-neuronal cells (Figure 6C). DEGs comparing PCB-exposed and non-exposed conditions for each broad cell type in both human and mouse datasets were identified (Figure 6D). Strikingly, GABAergic and glutamatergic neurons in RTT cortices of both species exhibited a predominance of downregulated PCB-associated DEGs, suggesting conserved mechanisms of PCB-associated transcriptional repression in these neuronal populations. In contrast, non-neuronal cells in both human and mouse show a trend of upregulated DEGs, indicating a shared activation of transcriptional programs in these cell types following PCB exposure (Figure 6D). The top KEGG pathways enriched in human and mouse datasets were identified (Figures 6E and 6F, respectively). Both species exhibit dysregulation in pathways related to synaptic function, neurodegeneration, and metabolic regulation. Notably, conserved pathways include synaptic signaling such as GABAergic synapse and glutamatergic synapse and retrograde endocannabinoid signaling in addition to metabolic and addiction pathways (Figures 6E and 6F). Interestingly, the female-specific noncoding *XIST* gene was significantly downregulated in response to PCB exposure in human cortex and showed concordant downregulation in mouse (*Xist*), demonstrating cross-species conservation of this effect. A highly significant interaction between *Mecp2e1* genotype and PCB exposure was observed for *Xist* levels, with cell type-specific differences (Figure S4). These findings underscore the translational relevance of our mouse model to human RTT, demonstrating conserved molecular and cellular responses to PCB exposure in *Mecp2* mosaic female cortex across species. The shared dysregulation of key pathways involved in synaptic function, neurodegeneration, and metabolic homeostasis highlights potential mechanistic links between an environmental exposure and RTT pathology.

**Figure 7:**
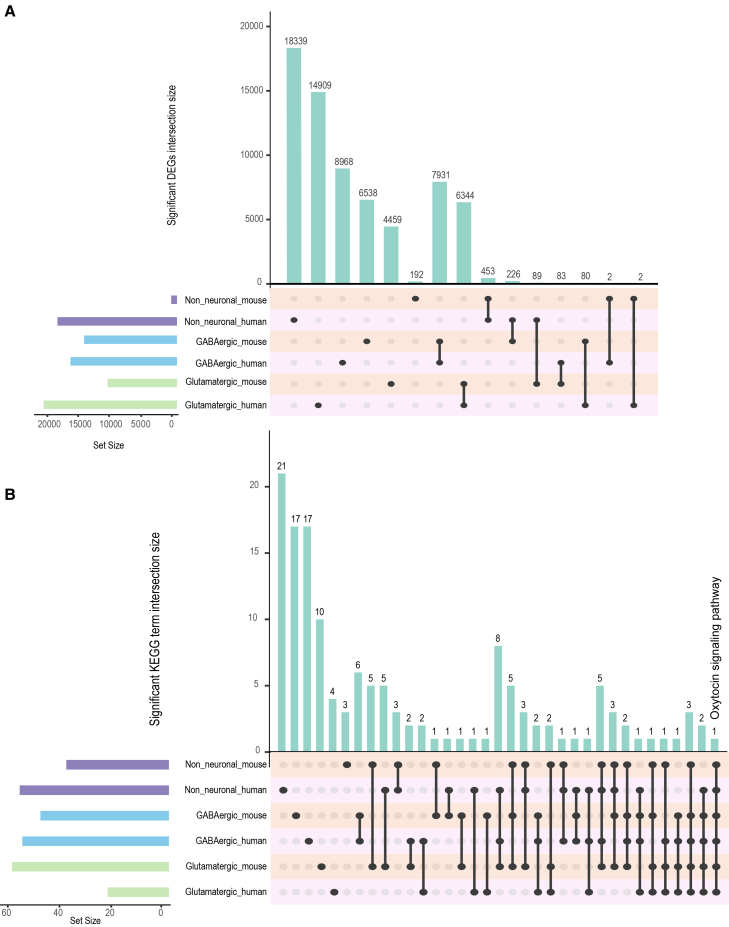
Cross-species conservation of PCB-associated transcriptional changes (A) UpSet plot showing the intersection of significant DEGs (adjusted *p* value ≤ 0.05) between human postmortem brain and mouse cortex across broad cell type categories. Set sizes (left) indicate the total number of significant DEGs in each species and cell type; intersection sizes (top) show the number of orthologous genes dysregulated in both species. (B) UpSet plot showing the intersection of significantly enriched KEGG pathways (adjusted *p* value ≤ 0.05) between human and mouse across broad cell types.

We further explored the conservation of molecular responses between mouse and human RTT cortex by analyzing the overlap of significant DEGs and KEGG pathways across GABAergic, glutamatergic, and non-neuronal cell types (Figure 7). The analysis of significant DEGs between mouse and human datasets reveals that the greatest overlap occurs in GABAergic cells (7,931 DEGs), followed by glutamatergic cells (6,344 DEGs), and lastly, non-neuronal cells (453 DEGs) (Figure 7A). Furthermore, we compared these conserved human-mouse DEGs with MeCP2 transcriptional target genes from published studies (RNA-Seq_Disease_Gene_and_Drug_Signatures database), resulting in significant enrichment (Fisher’s exact test, *p* = 3.78 × 10^−6^, OR = 1.29) (Figure S6; statistics and Mecp2 studies highlighted in Table S6). This pattern suggests that similar genes are dysregulated in these neuronal populations across species and different *Mecp2* mutant mouse models and brain regions, highlighting conserved transcriptional responses. The overlap of significant KEGG terms between mouse and human datasets across the three broad cell types were consistent with the DEG overlap. The greatest convergence of KEGG terms is observed in GABAergic cells, underscoring the shared molecular pathways affected in this cell type (Figure 7B). Notably, only one KEGG term, oxytocin signaling pathway, is common to both species and all three cell types, emphasizing oxytocin’s potential role as a conserved regulatory network of inter-cellular homeostatic responses in RTT.

These findings reinforce the translational relevance of this and other mouse models to human RTT, demonstrating conserved gene and pathway alterations, particularly affecting GABAergic and glutamatergic neurons. The identification of oxytocin signaling as a shared pathway across species and cell types suggests its potential importance in the pathophysiology of RTT.

## Discussion

This study provides a comprehensive analysis of how PCB exposure influences cortical cell type-specific gene expression in *Mecp2e1* mosaic females, shedding light on the complex interplay between an environmental factor and an X-linked gene, each regulating neurodevelopment. Using a combination of single-nucleus RNA sequencing, differential expression analysis, and network analysis, we explored the effects of PCBs on RTT syndrome transcriptional networks and identified potential inter-cellular interactions. We were able to directly distinguish WT- and mutant-expressing cells within the HET mosaic brains, facilitating a detailed comparative analysis of gene expression and pathway dysregulation between WT- and mutant-expressing neurons within and across experimental genotype and treatment groups.^21^ These experimental comparisons provided a high degree of specificity in parsing WT from mutant cells, which was crucial for understanding how PCB exposure impacts cell non-autonomous effects in the balance between inhibitory and excitatory neurons. The reduction in the number of RTT-associated dysregulated pathways observed with PCB exposure in the mouse model, combined with the substantial overlap of dysregulated KEGG pathways between mouse and human RTT cortices (Figure 7B), indicates a mitigating effect of PCB exposure on RTT-related gene dysregulation that is conserved across species. Together, these results provide multiple novel findings relevant to understanding the complex cellular and transcriptional pathogenesis of both RTT and PCB neurotoxicity.

Our prior analysis of RTT at the single-cell transcriptome level revealed that in HET female mice the WT-expressing neurons were dynamically responsive to the mosaic HET cortical environment.^21^ In the current study, both DEG and pathway analyses showed that PCB exposure led to fewer dysregulated genes compared to vehicle treatment in the RTT mutant mouse background. Parsing of the cells into mutant- and WT-expressing neurons demonstrated that PCB primarily impacted the WT-expressing cell transcriptome. This finding indicates a possible protective role of PCB exposure in the unusual mosaic context of RTT pathogenesis. The KEGG pathways commonly affected across all conditions included those relevant to adaptive pathways relevant to synaptic regulation, such as circadian rhythms and cAMP signaling, highlighting the broad impact of PCB exposure on critical homeostatic information networks between cells that are dysregulated in RTT.

We employed hdWGCNA to identify gene modules whose expression patterns are associated with *Mecp2* genotype and PCB exposure.^27^ We identified seven distinct gene modules, with multiple modules, including brown, that showed a significant positive correlation with *Mecp2* genotype in all cell types, but differed in the direction of correlations with PCB treatment. PCBs were negatively associated with brown module transcript levels predominantly in GABAergic neuronal subtypes. Interestingly, the female-specific long noncoding RNA *Xist* was a member of the brown network. Furthermore, there was a significant interaction between *Mecp2e1* genotype and PCB exposure that was cell type-dependent, with GABAergic neurons being the cell type with the strongest interaction effect. Somatostatin (Sst) cells, a GABAergic neuronal subtype, was found to be particularly strongly influenced by PCB exposure and *Mecp2e1* genotype. Together, these results suggest that *Xist* and other genes within the brown module may mediate the interactions between *Mecp2* genotype and PCB exposure in cortical GABAergic neurons. Future research could determine if interactions between PCBs and *Mecp2* are specific to females and if *Xist* is required for interaction effects in females.

While the study provides valuable insights, several limitations should be considered. Our analysis focused specifically on PCB exposure, but other environmental factors may also influence the inter-cellular transcriptional networks in the pathogenesis of RTT.^28^ Future research should explore a broader range of environmental exposures impacting RTT severity to gain a more comprehensive understanding. Longitudinal studies are needed to assess how gene expression changes evolve over time and their long-term implications for RTT and if different PCB doses change to effects on transcriptional and disease phenotypes.^29^ Additionally, further functional validation of the identified pathways and gene modules is necessary to confirm their roles in RTT and the impact of PCB exposure. While our study provides comprehensive transcriptional profiling of PCB-*Mecp2* interactions in a controlled mouse model with allele-specific resolution, there were limitations in the human samples. In the human postmortem validation cohort, we cannot separate *MECP2*-mutant from wild-type expressing cells due to the heterogeneity of mutations across individuals and technical constraints of 5′ snRNA-seq. Consequently, we cannot definitively rule out that subtle differences in X chromosome inactivation patterns between PCB-exposed and non-exposed individuals contribute to the observed transcriptional signatures. However, multiple lines of evidence support that the human findings reflect genuine biological responses to PCB exposure: our within-cell type analysis approach minimizes compositional confounding, the controlled mouse experiments demonstrate genuine PCB effects independent of XCI ratios, and most compellingly, we observe remarkable overlap of both DEGs and enriched KEGG pathways between the mouse and human datasets. This cross-species convergence at both gene and pathway levels, despite the inability to perform allele-specific analysis in human tissue, strongly suggests that the transcriptional signatures represent core, conserved PCB exposure effects that transcend individual *MECP2* mutation types and cellular mosaicism patterns.

While our study provides comprehensive transcriptional profiling with cross-species validation, we acknowledge that additional biological validation experiments (e.g., protein-level confirmation, functional electrophysiology, and behavioral phenotyping) would further strengthen these findings and are important directions for future mechanistic studies. The transcriptional atlas we provide establishes a foundation for hypothesis-driven validation of specific pathways and genes in future investigations. Future studies incorporating MeCP2 protein quantification, full-length RNA-seq approaches, or spatial transcriptomics with protein co-detection would enable more definitive assessment of cell-autonomous versus non-cell-autonomous PCB effects in human RTT brain tissue and could further validate these findings.

In conclusion, this study advances our understanding of how PCB exposure interacts with *Mecp2e1* mutations to influence RTT molecular pathogenesis in female mouse and human cortex. By elucidating the effects on gene expression, pathway dysregulation, and network dynamics, we could focus on therapeutic targets and mechanisms that could inform future research and clinical strategies for managing RTT. Our findings have several potential therapeutic implications for RTT management. First, the identification of oxytocin signaling as a conserved pathway across species and cell types (Figure 7B) suggests this system may represent a viable therapeutic target, particularly given recent success of combined oxytocin and IGF-1 in a mouse model of RTT.^30^ Second, the predominant effects of PCB exposure on WT-expressing neurons in the mosaic HET cortex suggest that therapies aimed at supporting wild-type cell function and cell-cell communication may be as important as those targeting mutant cells directly. Third, the dysregulation of GABAergic neuron-specific pathways, particularly in Sst interneurons, identifies specific cellular targets for intervention. The brown module genes, which show strong genotype-PCB interactions, may serve as biomarkers for environmental susceptibility and could inform personalized risk assessment. Finally, our findings that PCB exposure can mitigate certain RTT-associated transcriptional signatures suggest that understanding homeostatic compensation mechanisms in response to environmental perturbations may reveal endogenous protective pathways that could be pharmacologically activated. These molecular insights provide a foundation for hypothesis-driven therapeutic development and suggest that managing environmental exposures may be an important, yet underappreciated, component of comprehensive RTT care.

### Limitations of the study

This study captures gene expression at a single time point, and longitudinal studies are needed to assess how these changes evolve over time. The sample size, particularly for the mosaicism analysis, was limited, which may reduce statistical power to detect subtle allele-specific transcriptional differences between WT and mutant expressing cells within the mosaic HET cortex. In the human postmortem validation cohort, *MECP2* mutant and wild-type-expressing cells could not be separated due to the heterogeneity of mutations across individuals and technical constraints of 5′ snRNA-seq, meaning subtle differences in X chromosome inactivation patterns between PCB exposed and non-exposed individuals cannot be definitively ruled out as contributing to the observed transcriptional signatures. Additionally, this study assesses transcriptional changes without corresponding neurological or behavioral phenotyping, limiting our ability to directly link the observed gene expression and pathway dysregulation to functional outcomes in RTT. Future studies incorporating larger sample sizes, behavioral assessments, protein quantification, and spatial transcriptomics would strengthen and extend these findings.

## Resource availability

### Lead contact

Further information and requests for resources and reagents should be directed to and will be fulfilled by the lead contact, Janine M. LaSalle (jmlasalle@ucdavis.edu).

### Materials availability

This study did not generate new unique reagents.

### Data and code availability

• Raw and processed sequencing data have been deposited at GEO: GSE316011 and is publicly available as of the date of publication.
• All original code has been deposited at GitHub and is publicly available as of the date of publication (https://github.com/osmansharifi/PCB_mouse_snRNAseq).
• Any additional information required to reanalyze the data reported in this paper is available from the lead contact upon request.

## Acknowledgments

We would like to express our gratitude to the members of the Flow Cytometry Core, Bridget McLaughlin, and Jonathan Van Dyke for their technical assistance and expertise in sorting nuclei. This research was supported by 10.13039/100000002NIH T32ES007059, R01ES029213, R01AA027075, S10OD010786, U2CES030158, P50HD103526, and U24DK097154. The synthesis of the PCB mixture was supported by the Superfund Research Center at 10.13039/100008893The University of Iowa (P42 ES013661).

## Author contributions

O.S., K.E.N., and J.M.L. designed the study; J.M.L. and O.S. acquired funding for the study; K.E.N. and A.V. performed the PCB dosing and dissections of the mice; O.S. and D.H.Y. performed the nuclei isolation and single-cell assays; O.S. performed the bioinformatic analyses with intellectual contributions from I.K.; O.S. wrote the manuscript with intellectual contributions from K.E.N., P.J.L., D.H.Y., and J.M.L.; C.G.T. and O.S. generated all the figures. All authors reviewed and approved the final manuscript.

## Declaration of interests

Osman Sharifi, Dag H. Yasui, and Janine M. LaSalle are co-founders of 2C Bioscience Inc. The remaining authors declare no competing interests.

## Declaration of generative AI and AI-assisted technologies in the writing process

During the preparation of this work, the authors did not use generative AI tools and take full responsibility for the content of the published article.

## STAR★Methods

### Key resources table

**Table:** 

REAGENT or RESOURCE	SOURCE	IDENTIFIER
**Biological samples**		

Mouse cortical tissue from *Mecp2e1*^*−/+*^ and WT female mice	This paper	Mecp2e1^−/+^
Human postmortem RTT cortex samples with measured PCB levels	Sharifi et al.^21^	GEO: GSE211785

**Chemicals, peptides, and recombinant proteins**		

MARBLES PCB mixture (12 congeners: PCB 28, 11, 118, 101, 52, 153, 180, 149, 138, 84, 135, 95)	Prepared as in Neier et al.^15^	N/A
RNase inhibitor	Roche	N/A
DAPI	Fisher Scientific	N/A

**Critical commercial assays**		

Chromium Single Cell 5′ Library & Gel Bead Kit V1	10× Genomics	Cat#1000006
Kapa Library Quantification Kit	Roche	Cat#07960140001

**Deposited data**		

Raw and analyzed mouse snRNA-seq data	This paper	GEO: GSE316011
Analysis code	This paper	https://github.com/osmansharifi/PCB_mouse_snRNAseq
Human RTT snRNA-seq data	Sharifi et al.^21^	GEO: GSE211785
Mouse reference genome mm10–1.2.0	10× Genomics/Genome Reference Consortium	https://support.10xgenomics.com/single-cell-gene-expression/software/release-notes/build
Allen Brain Atlas mouse cortex reference	Allen Institute for Brain Science	https://portal.brain-map.org/
Allen Brain Atlas human cortex reference	Allen Institute for Brain Science	https://portal.brain-map.org/

**Experimental models: Organisms/strains**		

Mouse: *Mecp2e1*^*−/+*^: C57BL/6	LaSalle Lab; Sharifi et al.^21^	N/A
Mouse: C57BL/6J (wild-type)	The Jackson Laboratory	JAX: 000664; RRID: IMSR_JAX:000664

**Software and algorithms**		

Cell Ranger v3.2.3	10× Genomics	https://support.10xgenomics.com/single-cell-gene-expression/software/pipelines/latest/what-is-cell-ranger
R v4.2.2	R Foundation	https://www.r-project.org/
Seurat v4.3.0.1	Satija Lab	https://satijalab.org/seurat/; RRID: SCR_016341
scCustomize v2.1.1	Marsh et al.^31^	https://samuel-marsh.github.io/scCustomize/
LimmaVoom	Ritchie et al.	https://bioconductor.org/packages/limma/; RRID: SCR_010943
enrichR v3.2	Kuleshov et al.^32^	https://maayanlab.cloud/Enrichr/; RRID: SCR_001575
hdWGCNA v0.2.4	Morabito et al.^27^	https://smorabit.github.io/hdWGCNA/
abBLAST v3.0	Gish Lab	https://blast.advbiocomp.com/
BWA v0.7.17	Li and Durbin	https://bio-bwa.sourceforge.net/; RRID: SCR_010910
alleler.py (Mecp2e1 allele parsing pipeline)	Sharifi et al.^33^	https://github.com/osmansharifi/PCB_mouse_snRNAseq
biomaRt v3.21	Durinck et al.	https://bioconductor.org/packages/biomaRt/; RRID: SCR_019214
VennDiagram v1.7.3	Chen and Boutros	https://cran.r-project.org/package=VennDiagram
UCell	Andreatta and Carmona	https://bioconductor.org/packages/UCell/

**Other**		

70 μm FlowMi cell strainer	SP Scienceware (Bel-Art)	N/A
35 μm FlowMi cell strainer	SP Scienceware (Bel-Art)	N/A
MoFlo Astrios cell sorter	Beckman Coulter	N/A
Countess cell counter	Fisher Scientific	N/A
NovaSeq S4 sequencer	Illumina	N/A

### Experimental model and study participant details

#### Mouse dosing

*Mecp2e1*^*-/+*^ female mice were crossed with wild-type C57BL6/J males (Jackson Labs strain 000664) to generate the *Mecp2e1*^*-/+*^ progeny used in this study. This breeding strategy follows the established model^34^ producing females with mosaic expression of MeCP2-e1 due to random X-chromosome inactivation. A PCB mixture modeling the 12 most prevalent congeners found in maternal serum from the ASD-enriched MARBLES cohort was prepared according to previously established protocols.^12^^,^^15^ This formulation contained varying proportions of PCB congeners: PCB 28 (48.2%), PCB 11 (24.3%), PCB 118 (4.9%), PCB 101 (4.5%), PCB 52 (4.5%), PCB 153 (3.1%), PCB 180 (2.8%), PCB 149 (2.1%), PCB 138 (1.7%), PCB 84 (1.5%), PCB 135 (1.3%), and PCB 95 (1.2%). Female C57BL/6J mice (The Jackson Laboratory), ∼10 weeks of age, were administered either 0.01 mg/kg/day of the PCB mixture delivered via diet (in peanut butter) or vehicle control (peanut oil in peanut butter) for about 6 weeks. On week ∼16, dams (n_WT PCB-exposed_ =4, n_*Mecp2-e1*_ _PCB-exposed_ =4, n_WT vehicle control_ =4, n_*Mecp2-e1*_ _vehicle_ =4) were euthanized. From a total of 16 dams, cerebral cortices were harvested and used for single nucleus preparations. All experimental procedures received approval from the University of California, Davis Institutional Animal Care and Use Committee (IACUC #20584).

### Method details

#### Single-nucleus RNA sequencing

Single nucleus suspensions were prepared from each mouse brain according to previous stablished protocols.^21^^,^^35^ A 3.0 mm^2^ section of cortex was excised from each mouse brain and minced using a scalpel and subsequently homogenized in 0.5 ml of nuclei lysis buffer containing RNAse inhibitor (Roche, Indianapolis, ID). The homogenate was transferred to a larger vessel with an additional 1.0 ml of nuclei lysis buffer, thoroughly mixed, and incubated on ice for 5 minutes. Nuclei were separated from the lysate by filtration through a 70 μm FlowMi cell strainer (Sp-Belart, Wayne, NJ).

The nuclei were then pelleted by centrifugation at 500×G for 5 minutes at 4°C and resuspended in 1.5 ml of nuclei wash buffer for a 5-minute incubation. Following this incubation, nuclei were pelleted again under identical centrifugation conditions and washed twice in nuclei wash and resuspension buffer. The suspension was filtered through a 35 μm FlowMi filter (Sp-Belart, Wayne, NJ) and resuspended in nuclei wash and resuspension buffer containing 5 μg/ml DAPI. Nuclear concentration and integrity were assessed using a Countess cell counter (Fisher Scientific, Waltham, MA).

To remove debris and nuclear aggregates, the samples were processed on a MoFlow Astrios cell sorter (Beckman-Coulter, Brea, CA). Approximately 150,000 nuclei per sample were sorted and maintained on ice prior to library generation.

Single Cell 5′ Library V1 & Gel Bead Kits (10× Genomics, Pleasanton, CA) were employed for cDNA preparation and generation of barcoded and indexed snRNA-seq 5′ libraries following the manufacturer's protocol. A target of 10,000 nuclei per sample was established. The snRNA-seq 5′ libraries were quantified using a Kapa library quantification kit (Roche, Indianapolis, IN) and subsequently pooled for sequencing. Paired-end sequences of 150 base pairs were generated using a NovaSeq S4 sequencer (Illumina, San Diego, CA). The mouse cortical samples yielded approximately 75,000 reads per cell and an average of 240,437,728 reads per sample.

### Quantification and statistical analyses

#### Bioinformatic analyses

Raw reads from mouse samples were aligned to the mm10-1.2.0 reference genome using Cellranger v.3.2.3. The resulting cell by gene count matrices were imported into R 4.2.2 to create Seurat objects using Seurat_4.3.0.1. Quality control filtering was applied to mouse samples using the following criteria: cells were required to have less than 7% mitochondrial content, between 200 and 5,625 expressed genes, and between 208 and 16,300 unique molecular identifiers (UMI). The expression counts were log-transformed, normalized, and scaled using Seurat 4.3.0.1. Sample metadata was enriched with information regarding genotype, treatment, and *Mecp2-e1* allele status. For cell type annotation, previously published single nucleus data from RTT mouse cortex served as a ref.^21^ Cell type labels were transferred to the current dataset using scTransform for alignment. Cell marker testing was performed to validate the accuracy of cell type annotations. Visualization tools including UMAP plots and dotplots demonstrating validation of cell type-specific markers were generated using scCustomize 2.1.1.^31^

LimmaVoom was employed to identify differentially expressed genes (DEGs) across 15 distinct experimental comparisons. Prior to differential expression analysis, cell numbers were normalized by down sampling to match the condition with the fewest cells within each experiment. Complete parameters for all differential expression analyses are available in the GitHub repository. DEGs with a *p*-value ≤0.05 from each experiment were subsequently used as input for KEGG enrichment. For visualization of top DEGs in heatmaps (Figures 2B and 2C), we filtered for significant genes (adjusted *p*-value ≤ 0.05) within each cell type and comparison, then ranked by absolute log fold change. The highest-ranking genes from each comparison category were selected for display. KEGG pathway analysis was performed using the enrichR 3.2 R package with the default background gene set, consisting of all genes annotated in the KEGG database for mouse or human as appropriate. Fisher's exact test was used to determine enrichment significance, with Benjamini-Hochberg correction applied for multiple testing (adjusted *p*-value ≤ 0.05 considered significant).

To evaluate overlap between conserved PCB-responsive DEGs and known MeCP2 transcriptional signatures, enrichment analysis was performed using the RNA-Seq_Disease_Gene_and_Drug_Signatures database via enrichR 3.2.^32^ Enrichment terms containing “MeCP2” were extracted from results across all three cell types (GABAergic, Glutamatergic, and Non-neuronal), and all associated genes were compiled into a unified gene set (Table S6). This MeCP2 gene set was compared against the union of cell-type-matched human-mouse DEG intersections (genes differentially expressed in both species within each cell type). Statistical significance of overlap was assessed using Fisher's Exact Test (two-sided) with a background of 30,000 protein-coding genes. The odds ratio and 95% confidence interval were calculated to quantify the direction and magnitude of association, where an odds ratio < 1 indicates significant depletion and an odds ratio > 1 indicates significant enrichment (Figure S6). Overlap visualization and statistical analysis were performed using VennDiagram 1.7.3 in R.

For hdWGCNA analysis, cells from all four experimental conditions (WT_Vehicle, WT_PCB, HET_Vehicle, and HET_PCB) in the processed Seurat object were utilized as input to construct gene co-expression modules. Additional covariates included pregnancy status, weight at euthanasia, and treatment exposure duration. Genes were required to be expressed in at least 5% of cells to be included in the analysis. A signed network with a softpower of 0.8 was employed, resulting in the identification of 7 modules. Module scores were computed using the UCell method. Module-trait correlations were calculated using Pearson correlation between module eigengene values and experimental variables (PCB treatment and genotype) for each cell. Significance was determined using correlation tests with Benjamini-Hochberg correction for multiple comparisons (adjusted *p*-value ≤ 0.05). Results are provided in Figure S3 and Table S5. Standard pipelines from hdWGCNA 0.2.4 were followed, with detailed parameters available in the GitHub repository.

Parsing *Mecp2e1* alleles in the mosaic cortices was performed based on a previously published method.^33^ *Mecp2* reads were extracted from the raw fastq files generated for each sample. abBLAST 3.0 and BWA 0.7.17 mem were used in combination to extract Mecp2 reads (via alleler.py). The reference for alignment consisted of 100 bp of the *Mecp2* gene, comprising 50 bp upstream and 50 bp downstream of the exon1 start codon. Within the aligned reads, *Mecp2e1* mutant (TTG) and wild-type (ATG) start codons were enumerated using alleler.py. Each read contained cell barcode and UMI information, which enabled the incorporation of mutant and wild-type cell information into the Seurat object metadata.

For differential expression analysis, each gene in each sample was modeled using a negative binomial distribution. The mean was calculated as the product of library size and relative abundance (gene expression levels), with variance as a function of the mean. Significant DEGs were identified based on an adjusted *p*-value ≤0.05, employing the Benjamini-Hochberg method to control for false discovery. These significant DEGs served as input for KEGG pathway analysis, which utilized Fisher's exact test to determine if the overlap between input DEGs and background genes was significant (adjusted *p*-value ≤0.05). Cross-species ortholog mapping between human and mouse was performed using the biomaRt R package (version 3.21) to access the Ensembl ortholog database. For each human gene identified as differentially expressed in PCB-exposed versus non-exposed postmortem brain tissue, we queried for the corresponding mouse ortholog. One-to-one orthologs were directly mapped. For one-to-many orthology relationships (one human gene with multiple mouse orthologs), we selected the primary ortholog designated by Ensembl based on sequence homology and synteny conservation. For many-to-one relationships, each human gene was independently mapped to the corresponding mouse ortholog. Genes lacking clearly defined Ensembl orthologs were excluded from cross-species analyses. The overlap between human and mouse DEGs and KEGG pathways was visualized using UpSet plots to identify conserved transcriptional responses to PCB exposure across species.

## Supplemental information

Document S1. Figures S1–S6

Table S1. Cellular proportions per cell type per experimental condition Values represent cell type proportions (%) within each experimental condition.

Table S2. List of significant KEGG terms per DEG test for each cell type

Table S3. Number of significant DEGs per DEG experiment for GABAergic, glutamatergic, and non-neuronal cells

Table S4. List of significant DEGs per cell type for 15 different DEG experiments

Table S5. Module trait correlations for hdWGCNA also reflected in Figure S3

Table S6. Seq_Disease_Gene_and_Drug_Signatures database for GABAergic, glutamatergic, and non-neuronal cell types Table includes term names, overlap statistics, *p* values, adjusted *p* values, odds ratios, combined scores, associated genes, and cell type annotations. Terms containing “MeCP2” were used to compile the MeCP2 transcriptional target gene set for overlap analysis with conserved human-mouse DEGs (Figure S6).
