# Supplementary material for: Female cortical cellular mosaicism underlies shared MeCP2 and PCB impacted gene pathways
Source: iScience. 2026 Apr 20;29(5):115573. doi: 10.1016/j.isci.2026.115573 (PMC13198081; doi:10.1016/j.isci.2026.115573)
Supplement: Document S1. Figures S1–S6 [file mmc1.pdf]

iScience, Volume *29*

Supplemental information

Female cortical cellular mosaicism underlies shared MeCP2 and PCB impacted gene pathways

Osman Sharifi, Kari E. Neier, Anthony Valenzuela, Christina G. Torres, Ian Korf, Pamela J. Lein, Dag H. Yasui, and Janine M. LaSalle

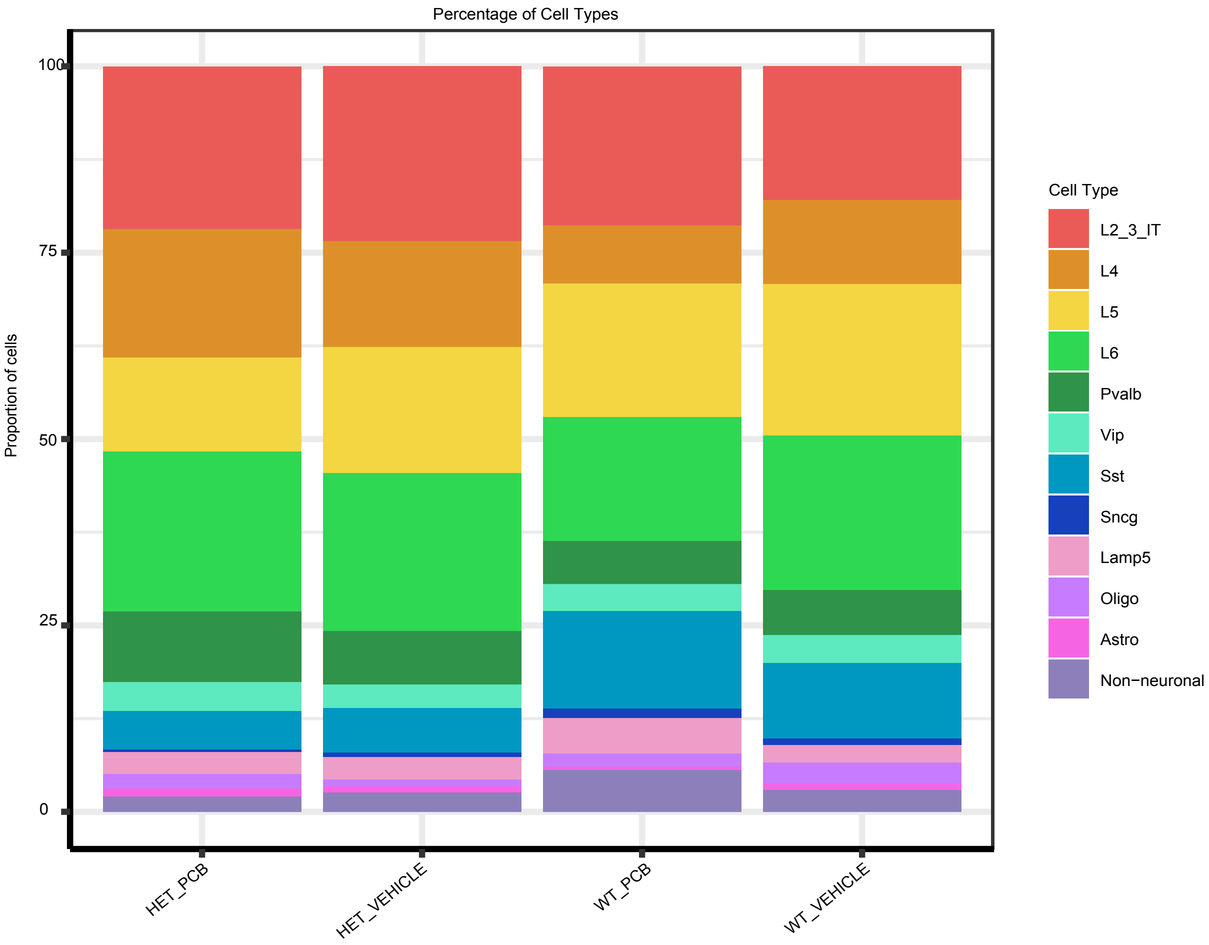

Supplemental Figure 1: Bar graph showing proportion of cell types across the four experimental groups.

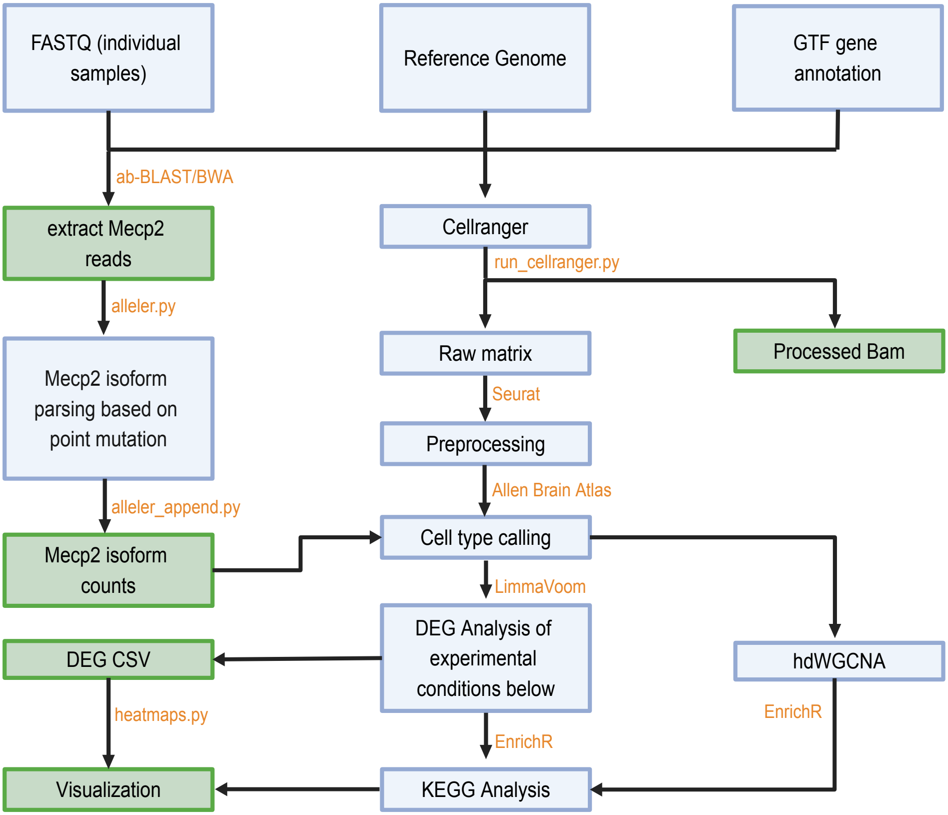

Supplemental Figure 2: Bioinformatic pipeline for parsing out Mecp2e1 WT and mutant-expressing cells in the mosaic RTT cortex and differential analysis.

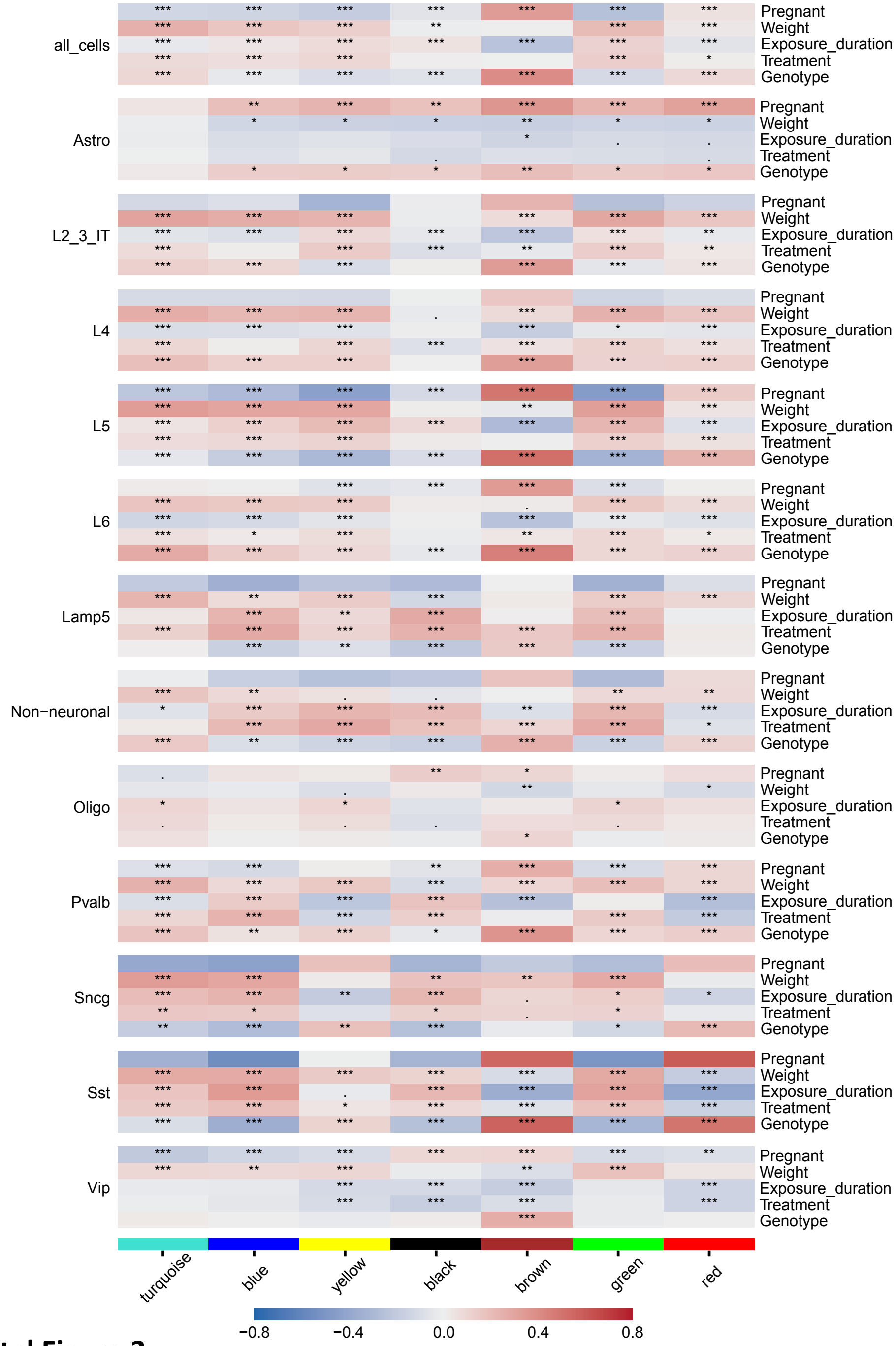

Supplemental Figure 3: Module-trait correlations for hdWGCNA analysis. Heatmap showing Pearson correlations between module eigengene values and experimental variables across cell types. Genotype: HET (Mecp2e1-/+) versus WT (Mecp2e1+/+); Treatment: PCB versus Vehicle; positive correlation values indicate higher expression in HET mice (for genotype) or PCB-exposed mice (for treatment). Exposure_duration: days of PCB exposure; Weight: body weight at sacrifice (~16 weeks); Pregnant: pregnancy status (binary). Significance levels: * adjusted p-value < 0.05, ** adjusted p-value < 0.01, *** adjusted p-value < 0.001.

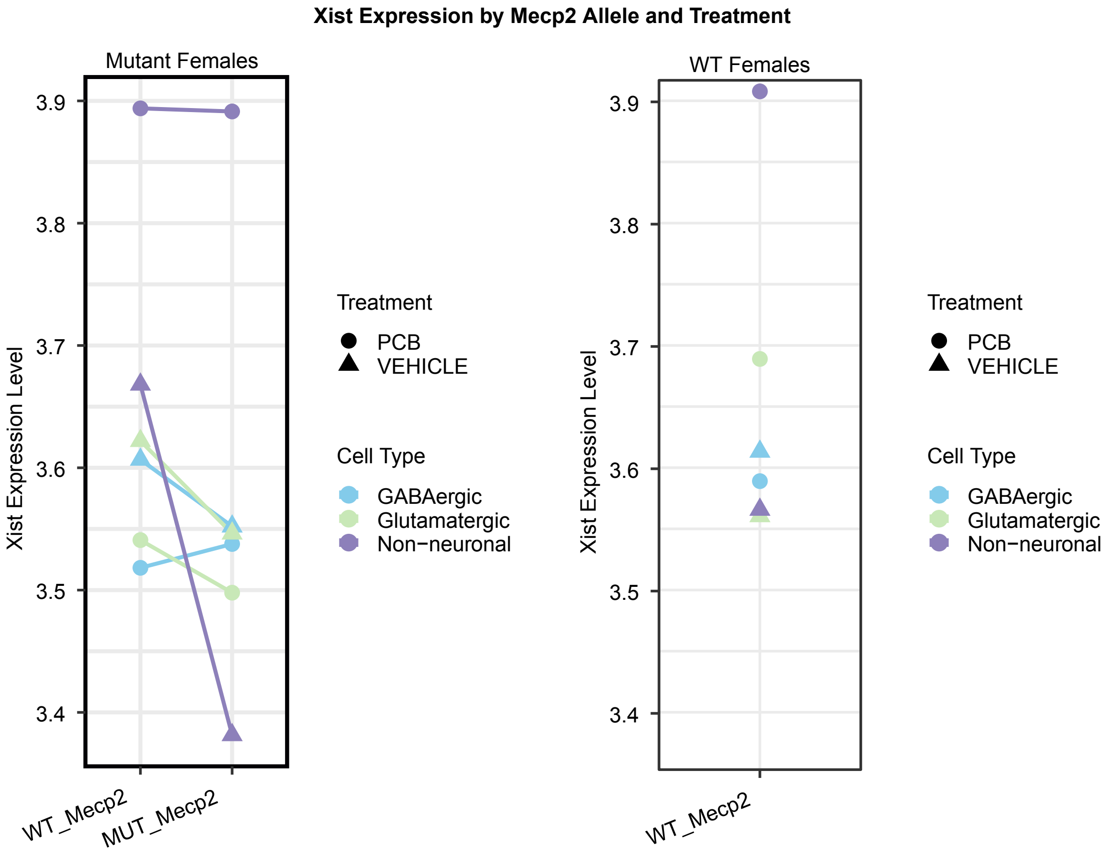

3-way ANOVA	Df	Sum Sq	Mean Sq	F value	Pr(>F)	Significance
Mecp2_allele	1	1.2	1.2216	3.608	0.0577	.
Treatment	1	0.5	0.538	1.589	0.2077	
Cell Type	2	0.3	0.1534	0.453	0.6357	
Mecp2_allele:Treatment	1	0.2	0.1967	0.581	0.446	
Mecp2_allele:Cell Type	2	0.3	0.1346	0.397	0.6721	
Treatment:Cell Type	2	2.3	1.1258	3.325	0.0363	*
Mecp2_allele:Treatment:Cell Type	2	0.2	0.0762	0.225	0.7986	
Residuals	1370	463.8	0.3386			

Signif. codes:	0 ‘***’	0.001 ‘**’	0.01 ‘*’	0.05 ‘.’	0.1 ' '	1

Supplemental Figure 4: Interaction plot of Xist expression with Mecp2e1 mutant allele, treatment and cell type.

PCB levels per human cortical sample

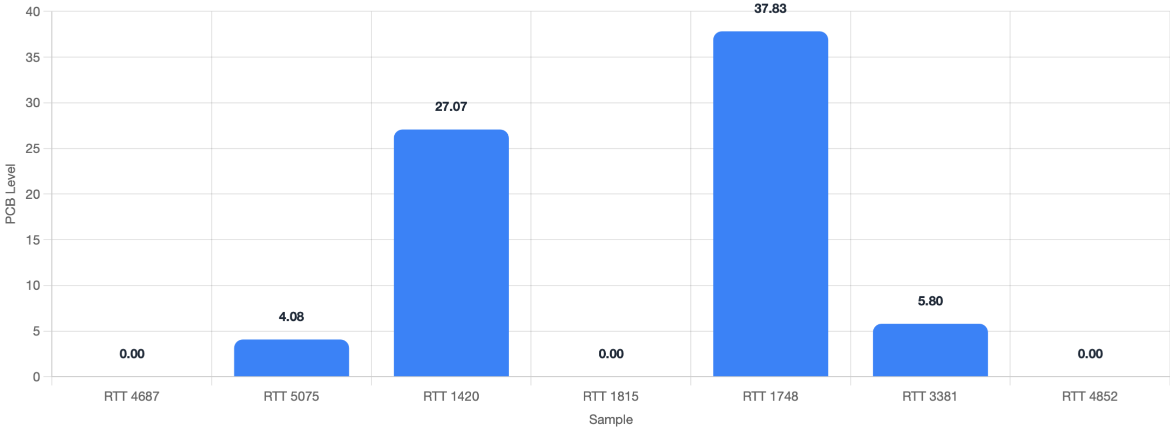

Supplemental Figure 5: PCB exposure levels for human samples measured as ng/g lipid.

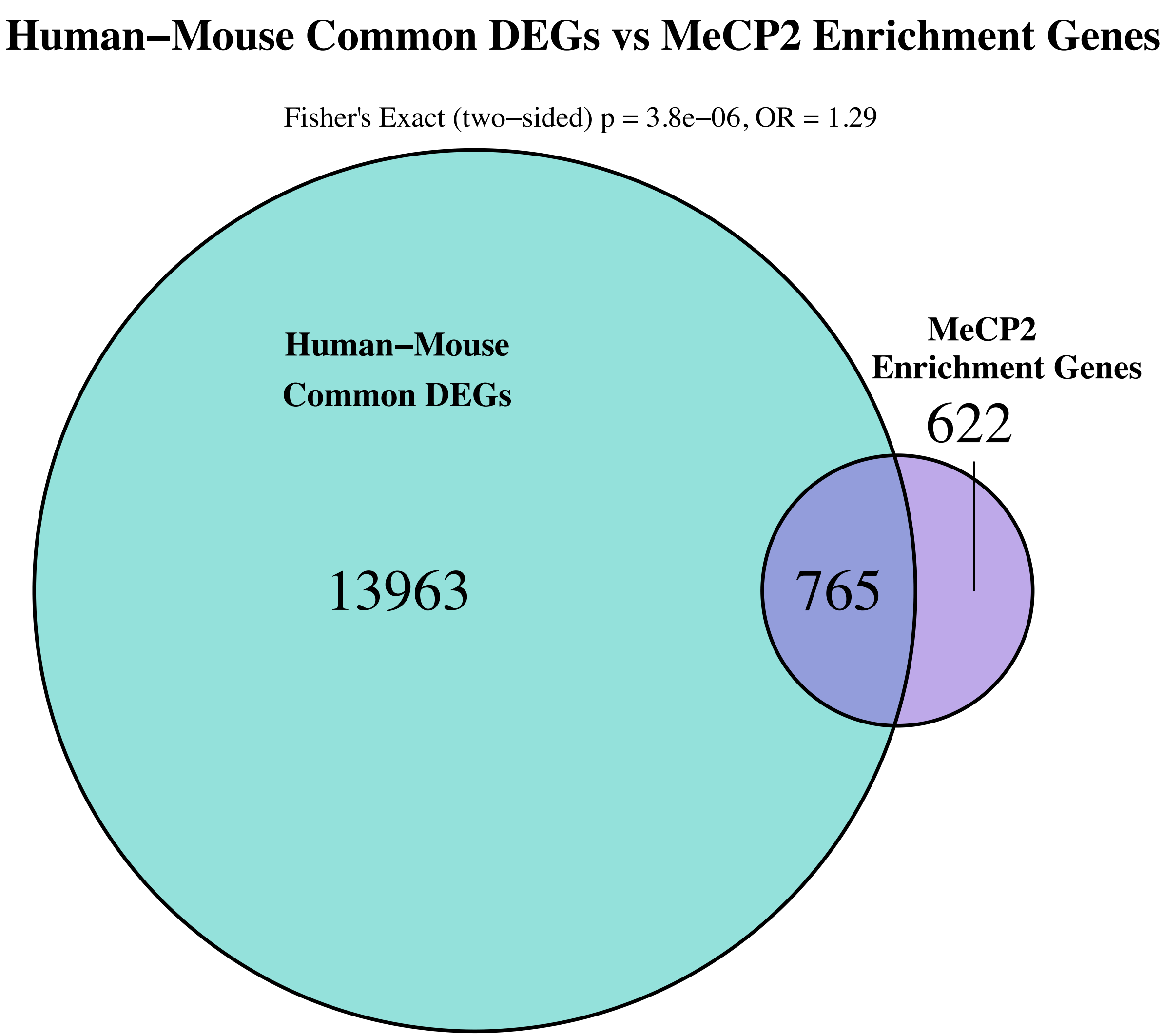

Supplemental Figure 6: Venn diagram showing overlap between conserved human-mouse PCB-responsive DEGs and MeCP2 transcriptional target genes. The human-mouse common DEGs represent the union of cell-type-matched intersections (genes differentially expressed in both species within GABAergic, Glutamatergic, and Non-neuronal populations). MeCP2 enrichment genes were derived from MeCP2-related terms in the RNA-Seq_Disease_Gene_and_Drug_Signatures database. Statistical significance was assessed using Fisher's Exact Test (two-sided) where OR is odds ratio.
